# Nanovirus Disease Complexes: An Emerging Threat in the Modern Era

**DOI:** 10.3389/fpls.2020.558403

**Published:** 2020-11-19

**Authors:** Aamir Lal, Thuy Thi Bich Vo, I Gusti Ngurah Prabu Wira Sanjaya, Phuong Thi Ho, Ji-Kwang Kim, Eui-Joon Kil, Sukchan Lee

**Affiliations:** ^1^Department of Integrative Biotechnology, Sungkyunkwan University, Suwon, South Korea; ^2^Research and Development Bureau, Chungcheongnam-do Agricultural Research and Extension Services, Yesan, South Korea; ^3^Department of Plant Medicals, Andong National University, Andong, South Korea

**Keywords:** nanoviruses, multipartite virus, evolution, host range, geographical distribution, geminiviruses

## Abstract

Multipartite viruses package their genomic segments independently and mainly infect plants; few target animals. *Nanoviridae* is a family of multipartite single-stranded DNA plant viruses that individually encapsidate single-stranded DNAs of approximately 1 kb and transmit them through aphids without replication in the aphid vectors, thereby causing important diseases of leguminous crops and banana. Significant findings regarding nanoviruses have recently been made on important features, such as their multicellular way of life, the transmission of distinct encapsidated genome segments through the vector body, evolutionary ambiguities, mode of infection, host range and geographical distribution. This review deals with all the above-mentioned features in view of recent advances with special emphasis on the emergence of new species and recognition of new host range of nanoviruses and aims to shed light on the evolutionary linkages, the potentially devastating impact on the world economy, and the future challenges imposed by nanoviruses.

## Introduction

Among viruses, single-stranded (ss) DNA viruses are a considerable threat to all living organisms. These ssDNA viruses infect both plants and animals. Circoviruses (Tischer et al., 1986; Cheung, 2006), bidensoviruses (Hayakawa et al., 2000), small circular (smaco) viruses (Ng et al., 2015), redondoviruses (Abbas et al., 2019), anelloviruses (Biagini et al., 2006; Blatter et al., 2018), genomoviruses (Krupovic et al., 2016), and circular replication-associated protein (Rep)-encoding single-stranded (CRESS) DNA viruses (Dayaram et al., 2014; Rosario et al., 2015) are some of the important ssDNA viruses, which infect animals, silkworm, human beings, fungi, insects and marine invertebrates, respectively. ssDNA viruses are largely known for their devastating effects on the plant world (Goodman, 1977; Kenyon et al., 2014; Malathi and Renuka Devi, 2019b). Among these ssDNA viruses, monopartite and bipartite viruses, with one and two segments, respectively, are very common. In these viruses, nucleic acid segments are encapsidated into a single virion (viral particle) which propagates as a whole. Some viruses are multipartite and have two or more segmented genomes packaged into separate virions, each of them capable of propagating independently (Randles et al., 2000; Sicard et al., 2016).

Based on their genomic organization, the International Committee on the Taxonomy of Viruses (ICTV) categorized ssDNA plant viruses into two families: (i) *Geminiviridae* (Zerbini et al., 2017) and (ii) *Nanoviridae* (Randles et al., 2000; Vetten et al., 2012). *Geminiviridae* is the largest family of plant viruses which can infect a large number of hosts belonging to several plant genera and families. *Nanoviridae*, the focal point of this study, comprises plant viruses possessing very small virions which contain a multipartite (6–8), circular, single stranded DNA genome of approximately 1 kb in length, along with a few satellite molecules, each possessing a specific function (Vetten et al., 2012; Briddon et al., 2018; Malathi and Dasgupta, 2019a). Highly diversified host ranges are ascribed to *Nanoviridae* members which induce symptoms such as stunting, dwarfism, necrosis, mosaic, and leaf rolling in host plants and may eventually lead to plant death as well (Mandal, 2010; Grigoras et al., 2014; Hull, 2014; Gaafar et al., 2017, 2018). Viral replication occurs in the nucleus of infected cells via ssDNA rolling circle amplification (Rosario et al., 2012; Jeske, 2018). In addition, aphid transmission is a key characteristic for viruses belonging to the *Nanoviridae* family (Sano et al., 1998; Franz et al., 1999). Here we outline the *Nanoviridae* family and delve into the recent developments while identifying its impact on the agricultural world.

## *Nanoviridae* Family: Classification, Genomic-Structure, and Function

**FIGURE 1:**
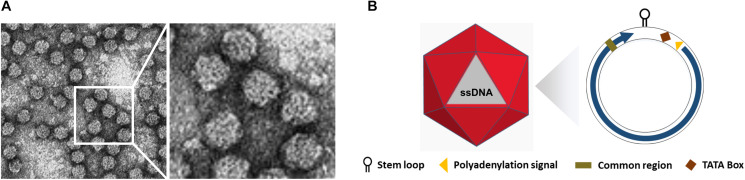
Structure of Nanoviruses. **(A)** Negative contrast electron micrograph of particles of Faba bean necrotic yellows virus (FBNYV). The bar represents 50 nm. (Courtesy of L. Katul and D.-E. Lesemann.) **(B)** Non-enveloped ssDNA with icosahedral and round geometries, and T = 1 symmetry. The diameter is around 18–19 nm. The encoded protein (ORF) is indicated inside circles by arrow.

The *International Committee on Taxonomy of Viruses (ICTV)* categorized the *Nanoviridae* family into two genera, *Nanovirus* and *Babuvirus*, based on their genome organization and transmission vectors, along with categorization of coconut foliar decay virus (CFDV) as an unassigned species (Mandal, 2010; Table 1). Nanoviruses are non-enveloped with icosahedral and round geometries, and *T* = 1 symmetry with a diameter of 18–19 nm (Figures 1A,B). Contrary to geminiviruses, nanoviruses are multipartite viruses with 8–10 circular ssDNA components of approximately 1 kb in size (Sano et al., 1998; Gronenborn, 2004), while babuviruses contain six components with a size of approximately 1–1.1 kb (Halbert and Baker, 2015) and 12 DNAs of approximately 1.2–1.4 kb in size in association with CFDV (Gronenborn et al., 2018). Additional circular, ssDNA molecules (∼1–1.4 kb) that encode Rep protein, referred to as satellite molecules, have also been reported along with nanoviruses and babuviruses recently and categorized as *nano alphasatellites*. Those in the unassigned CFDV are categorized into unassigned species in the family *alphasatellitidae* (Briddon et al., 2018).

**TABLE 1:** Introduction to *Nanoviridae*: occurrence, transmission, host ranges and symptoms development.

Genus	Species	Family	Host Species	Symptoms	Transmission	References
**Nanovirus**	*Subterranean clover stunt virus* (SCSV)	*Fabaceae*	*Trifolium subterraneum Medicago hispida Macroptilium lathyroides*	Chlorosis and stunting	*Aphis craccivora, A. gossypii*,	Boevink et al., 1995
	*Faba bean necrotic yellows virus* (FBNYV)	*Fabaceae*	*Cicer arietinum, Vicia faba Phaseolus vulgaris*	Necrosis and leaf rolling	*Acyrthosiphon pisum*	Katul et al., 1998
	*Faba bean necrotic stunt virus* (FBNSY)	*Fabaceae*	*Lens culinaris Vicia sativa*	Necrosis and stunting	*Acyrthosiphon pisum Aphis craccivora*	Grigoras et al., 2010a
	*Pea necrotic yellow dwarf virus* (PNYDV)	*Fabaceae*	*Pisum sativum Vicia faba, V. sativa* and *Lens culinaris*	Stunting, dwarfing, yellowing and leaf rolling	*Acyrthosiphon pisum*	Gaafar et al., 2016
	*Milk Vetch Dwarf Virus* (MDV)	*Fabaceae Caricaceae Solanaceae*	*Astragalus sinicus Glycine max Carica papaya Solanum lycopersicum Capsicum annuum*	Stunting, dwarfing, vein yellowing	*Aphis craccivora*	Sano et al., 1998
	*Faba bean yellow leaf virus* (FBYLV)	*Fabaceae*	*Vicia faba*	Yellowing, stunting, necrosis and leaf deformation	*Acyrthosiphon pisum*	Abraham et al., 2012
	*Black medic leaf roll virus* (BMLRV)	*Fabaceae*	*Medicago lupulina Pisum sativum*	Leaf rolling	*–*	Grigoras et al., 2014
	*Pea yellow stunt virus* (PYSV)	*Fabaceae*	*Pisum sativum*	Stunting, yellowing	*–*	Grigoras et al., 2014
	*Cow vetch latent virus* (CvLV)	*Fabaceae*	*Vicia cracca*	*–*	*–*	Gallet et al., 2018
	*Sophora yellow stunt associated virus* (SYSaV)	*Fabaceae*	*Sophora alopecuroides L.*	Dwarfing, yellowing, stunted leaves and yellow vein banding.	*–*	Heydarnejad et al., 2017
	*Parsley severe stunt associated virus* (PSSaV)	*Apiaceae*	*Petroselinum crispum (Mill.) Fuss*	Stunting, leaf yellowing and leaf curling.	*–*	Vetten et al., 2019
	*Milk vetch chlorotic dwarf virus* (MVCDV)	*Fabaceae*	*Astragalus myriacanthus Boiss.*	Leaf chlorosis, little leaves and dwarfism	*–*	Hassan-Sheikhi et al., 2020
**Babuvirus**	*Banana bunchy top virus* (BBTV)	*Musaceae*	*Musa* spp.	Dark green streaks plant stunting	*Pentalonia nigronervosa*	Burns et al., 1995
	*Abaca bunchy top virus* (ABTV)	*Musaceae*	*Musa* spp.	Mosaic	*–*	Sharman et al., 2008
	*Cardamom bushy dwarf virus* (CBDV)	*Zingiberaceae*	*Amomum subulatum*	Streak mosaic Bushy appearance	*Micromyzus-kalimpongensis*	Mandal et al., 2004
***Coconut foliar decay virus***	*Coconut foliar decay virus* (CFDV)	*Arecaceae*	*Cocos nucifera*	foliar decay	*Myndus tiffany*	Gronenborn et al., 2018

**FIGURE 2:**
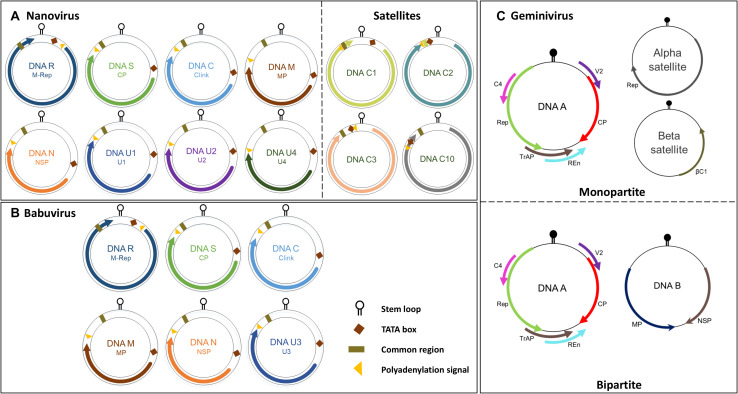
Genome organization of the nanovirus, babuvirus and geminivirus. **(A)** Nanovirus and babuvirus comprises of eight ssDNA components along with three or four satellite molecules. **(B)** Babuvirus comprises of six ssDNA components. In both nanoviruses and babuviruses, the name of each genome segment and the name of the encoded protein is indicated inside circles in respective colors: Clink, Cell-cycle linked protein; MP, movement protein; NSP, nuclear shuttle protein; M-Rep, master rep; CP, coat protein; U1, U2, U4 (nanovirus) and U3 (babuvirus). **(C)** Geminiviruses are categorized in monopartite and bipartite based on genome organization. Monopartite geminiviruses contains a main ssDNA (DNA A) component in which six ORFs are present which encodes specific proteins to perform different functions: replication-associated protein (Rep), coat protein (CP), replication enhancer protein (REn), transcriptional activator protein (TrAP), proteins involved in virus movement (AV2), pathogenicity determinant and a suppressor of RNA silencing (AC4). Monopartite geminiviruses contain alphasatellites which encode for Rep protein (Rep) or betasatellites which have a βC1 gene, satellite conserved region (SCR) or both. Bipartite viruses contain DNA B along with DNA A which encodes for MP and NSP. Stem loop, TATA box, common region and polyadenylation signal are also highlighted.

Whilst the genomes of geminiviruses are encoded by one or two circular ssDNA molecules, the genomes of Nanoviridae members are encoded by six or eight components (Figure 1B). Furthermore, these components are encapsidated separately into individual virions each with a specific role (Harrison, 1985; Randles et al., 2000; Saunders et al., 2003). DNA R encodes the master replication (M-Rep) initiator protein (Timchenko et al., 2000; Horser et al., 2001), DNA M encodes the movement protein, DNA C encodes the cell-cycle-link (clink) protein (Aronson et al., 2000), DNA S encodes the capsid protein (CP) (Wanitchakorn et al., 1997), and DNA N encodes the nuclear shuttle protein (NSP) (Wanitchakorn et al., 2000; Gronenborn, 2004) (Function of CP, Rep and NSP explained in following sections). Despite the numerous attempts to investigate U1, U2, and U4 of nanoviruses and U3 of babuviruses as well as the satellite molecules associated with nanoviruses, their biological functions remain unclear (Figures 2A–C).

## Geographical Distribution of *Nanoviridae* Members

**FIGURE 3:**
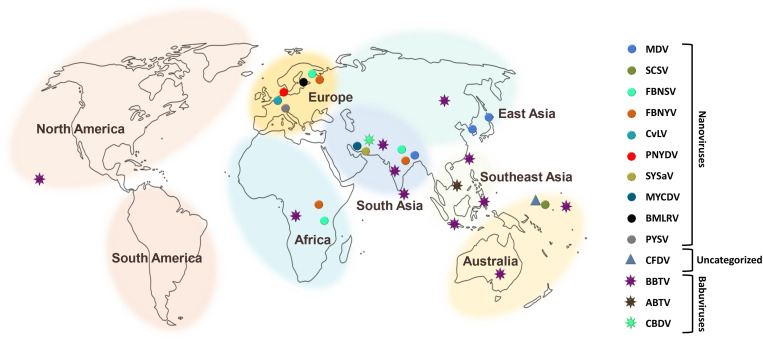
Geographical distribution of *Nanoviridae* members. Eight different centers at continent and subcontinent levels were marked: South America, North America, Africa, Europe, South Asia, Southeast Asia, East Asia, and Australia. Circles represent the species of the genus, *Nanovirus* whereas, species of the genus, *Babuvirus* are shown by the eight-point star. Triangle represents the uncategorized species i.e., *Coconut foliar decay virus*. To differentiate the species, circles, stars and triangles have been highlighted with different colors respectively.

There have been increasing reports of the presence of *Nanoviridae* members from different regions of the world. Between the genera of the family *Nanoviridae*, babuviruses are highly ubiquitous viruses, e.g., banana bunchy top virus (BBTV) has been reported almost throughout the world (Sun, 1961; Burns et al., 1995; Beetham et al., 1997; Amin et al., 2008; Almeida et al., 2009; Blomme et al., 2013). Abaca bunchy top virus (ABTV) and cardamom bushy dwarf virus (CBDV) are found in the Philippines and Malaysia (Sharman et al., 2008) and India, respectively (Mandal et al., 2004; Ghosh et al., 2015). *Nanoviridae* members have marked their presence in major continents: Asia, Europe, Africa, and Australia. Among these nanoviruses, some were observed to be limited to certain areas or particular countries within a continent, while some exhibited high diversity through their presence across many continents, such as cow vetch latent virus (CVLV) in France; sophora yellow stunt-associated virus (SYSaV) and milk vetch chlorotic dwarf virus (MVCDV) in Iran; and faba bean yellow leaf virus (FBYLV) reported only in Ethiopia (Abraham et al., 2012; Heydarnejad et al., 2017; Hassan-Sheikhi et al., 2020); milk vetch dwarf virus (MDV) and subterranean clover stunt virus (SCSV) in Asia (Boevink et al., 1995; Sano et al., 1998; Lal et al., 2018); pea necrotic yellow dwarf virus (PNYDV), black medic leafroll virus (BMLRV), parsley severe stunt associated virus (PSSaV) and pea yellow stunt virus (PYSV) in Europe (Grigoras et al., 2014; Gaafar et al., 2016; Vetten et al., 2019); faba bean necrotic yellows virus (FBNYV) and faba bean necrotic stunt virus (FBNSY) in Asia, Europe, and Africa (Katul et al., 1998; Grigoras et al., 2010b); and CFDV unassigned species detected from Vanuatu, located near the South Pacific Ocean (Gronenborn et al., 2018; Figure 3). Interestingly, no nanoviruses have been identified in the new world to date. Though some of these viruses could be invasive, while others may have been there for ages and have been increasingly identified in these regions due to an increasing number of metagenomic studies (Gaafar et al., 2018; Gronenborn et al., 2018; Vetten et al., 2019; Hassan-Sheikhi et al., 2020; Lal et al., 2020), data scarcity confines our analysis. The purpose of listing the species at different locations around the world is simply to reflect the regions in which the specific nanoviruses were identified.

## Host Range and Symptoms

Nanoviruses are considered as viral agents with limited host range. Among nanoviruses, babuviruses infect only the monocot species, *Musaceae* and *Zingiberaceae* (Burns et al., 1995; Mandal et al., 2004; Amin et al., 2008). No other plant families have been reported to be infected by babuviruses. BBTV mainly infects *Musa acuminata*, *M. coccinea*, *M. balbisiana*, *M. ornata*, *M. jackeyi*, *M. textilis*, and *M. velutina* (Burns et al., 1995; Sharman et al., 2008; Qazi, 2016). Nanoviruses were considered to affect only the legumes (Franz et al., 1997; Abraham et al., 2010; Grigoras et al., 2010a). Fabaceae, also known as Leguminosae, a legume family, is an ideal target for infection by nanovirus members (Abraham et al., 2010, 2012; Grigoras et al., 2014). About 50 Fabaceae species are infected by these members, and this number continues to increase (Franz et al., 1997; Gaafar et al., 2016) (nanoviruses with respective host ranges are listed in detail in Table 1). Nanoviruses limitation to narrow host ranges was a major factor in considering them as low impact viruses with an exiguous domain. This is why geminiviruses with the infection severity and outbreaks in broad host range have always been a preferred research area among ssDNA viruses compared to nanoviruses (Harrison, 1985; Mansoor et al., 2003; Jeske, 2009; Kenyon et al., 2014; Kil et al., 2016; Rodrigues et al., 2019). Recent developments have contributed to the discovery of new nanovirus hosts by confirming their presence in various important plant families including both dicots as well as monocots. For example, MDV was recently reported in dicots families i.e., Caricaceae (*Carica papaya*) (Lal et al., 2018), Solanaceae (*Solanum lycopersicum, Capsicum annuum*) (Lal et al., 2020) and in monocots family i.e., Liliaceae (*Lilium candidum*) (Lal et al., 2018). Moreover, the unassigned species, CFDV has been reported in a monocot family i.e., Arecaceae (*Cocos nucifera*) (Gronenborn et al., 2018). Recent identification of new nanoviruses in new host plants is an intriguing aspect to be focused on. PSSaV was recently reported in Apiaceae [*Petroselinum crispum* (Mill.) Fuss] (Vetten et al., 2019) whereas, MVCDV was reported in Fabaceae (*A. myriacanthus Boiss*) (Hassan-Sheikhi et al., 2020). Recent discoveries show the ongoing surge of viral infection evidence in various new host plant species owing to the growing number of metagenomics studies.

**FIGURE 4:**
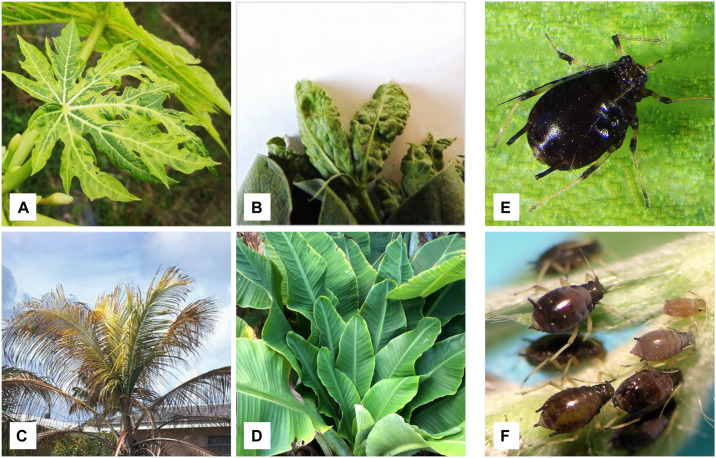
Symptomatic host plants of nanovirus and babuviruses along with insect vectors. **(A)** Papaya plant showing leaf yellowing and dwarfism and found infected with MDV (nanovirus). **(B)** Faba bean showing necrosis and infected with FBNYV (nanovirus). **(C)** Coconut tree exhibiting foliar decay due to CFDV (uncategorized). **(D)** Banana plant showing bunchy top disease symptoms and infected with BBTV. Insect vectors, **(E)** *Pentalonia nigronervosa* transmits babuviruses and **(F)** *Aphis craccivora* responsible for the transmission of nanoviruses.

Generally, symptom development in nanovirid-infected plant species resembles that observed in *Geminiviridae* infections, such as chlorosis, necrosis, leaf rolling, dwarfing, stunting, leaf yellowing, vein yellowing, leaf deformation, and plant death (Mansoor et al., 2003; Spence et al., 2007; Jeske, 2009; Hull, 2014; Kenyon et al., 2014; Gaafar et al., 2016; Rodrigues et al., 2019; Saucke et al., 2019; Vetten et al., 2019; Figures 4A–D). However, *Geminiviridae* has a much more diverse assortment of associated symptoms. *Nanoviruses* induce almost all of the symptoms mentioned above in their respective hosts (Table 1). Each nanovirus has been named according to its major symptom; for example, FBNYV, MVCDV, MDV, and BMLRV show leaf yellowing, leaf chlorosis, dwarfism, and leaf rolling, respectively. Babuviruses also show a slight deviation in symptom development, inducing dark green streaks, streak mosaicism and a bushy appearance in hosts infected by the BBTV, ABTV, and CBDV, respectively. Coconut foliar decay is observed in the case of unassigned species CFDV (Merits et al., 2000). Mostly, clear symptoms can be observed in parts of the plant infected by any member of the *Nanoviridae* family. but in few recently reported cases, no significant symptoms were observed i.e., MDV infection in Solanaceous members (Lal et al., 2020). Nanoviruses do not exhibit any phenotypic difference with other viruses and no symptom can be associated specifically with the nanoviruses to date.

## Divergence of *Nanoviridae* From *Geminiviridae* and *Circoviridae*

*Nanoviridae* are more closely related to *Geminiviridae* and *Circoviridae* among the seven families of ssDNA viruses in the phylum Cressdnaviricota (Krupovic et al., 2020). *Geminiviridae* is one of the largest families of plant viruses belonging to the order *Geplafuvirales* of the *Repensiviricetes* class. Replication occurs via a rolling circle mechanism, highly conserved sequences TARTATTAC (geminiviruses), TANTATTAC (nanoviruses) in the loop of a putative stem-loop structure within the IR, and the association with satellite molecules (especially alphasatellites in) are common features of the plant virus families *Geminiviridae* and *Nanoviridae* (Burns et al., 1995; Sano et al., 1998; Timchenko et al., 2000; Buchmann et al., 2009). Based on these resemblances, the members of these viral families are considered cousin viruses (Koonin et al., 2015).

The family *Circoviridae* involves a community of diverse animal viruses with small, closed-circular, ssDNA that belongs to the order *Cirlivirales* of the *Arfiviricetes* class. *Nanoviridae* shares the same class but different order i.e., Mulpavirales with *Circoviridae*. Their genome size is ∼1.7–2.1 kb and consists of two ORFs in the opposite direction with NANTATTAC as highly conserved sequences. These animal circoviruses are closely related to plant nanoviruses, as the Rep of circoviruses shows high similarity to the Rep of nanoviruses (Simmonds et al., 2017). The origin of replication (ori) in both the circovirus and nanovirus DNA is adjacent to the N-terminal part of the Rep gene (Niagro et al., 1998). This similarity between circovirus and nanovirus ori sequences indicates that these sequences have evolved from a common ancestral sequence (Katul et al., 1998), and that the circovirus has evolved to infect a vertebrate in various intermediate stages over time (Gibbs and Weiller, 1999). Another study showed that Rep proteins of marine ssDNA viruses show high resemblance with nanoviruses. A high copy number viral genome has been isolated from an algal cell identifying protists as the possible origin of nanoviruses, circoviruses and geminiviruses (Yoon et al., 2011). Despite some common factors, *Nanoviridae* exhibit certain contradictions to *Geminiviridae* and *Circoviridae* in terms of their genome organization, way of transmission, mode of infection, host range, and symptoms development (see details in Table 2; Sano et al., 1998; Gronenborn, 2004; Sharman et al., 2008; Iranzo and Manrubia, 2012; Halbert and Baker, 2015; Sicard et al., 2015; Di Mattia et al., 2020).

**TABLE 2:** Divergence of *Nanoviridae* from *Geminiviridae* and *Circoviridae*.

Type	Attributes	*Nanoviridae*		*Geminiviridae*	*Circoviridae*
		**Nanovirus**	**Babuvirus**	**Begomovirus**	**Circovirus**
**DNA**	Shape	Circular, ssDNA	Circular, ssDNA	Circular, ssDNA	Circular, ssDNA
	Partite	Multipartite (8–11 segments)	Multipartite (6 segments)	Monopartite or Bipartite	Monopartite
	ORFs	1 ORF in each component	1 or 2 ORFs in components	6–7 in DNA A 2 in DNA B	2 ORFs in opposite direction
	Stem loop (5’- 3)’	TANTATTAC	TATTATTAC	TATTATTAC	NANTATTAC
	Satellite molecules	∼3–4	1	2 (1 alpha and 1 Beta)	0
**Size**	Length	∼1 kb	∼1–1.1 kb	∼2.5–3 kb	∼1.7–2.1 kb
	Diameter	18–19 nm	17–20 nm	18–20 nm	∼20 nm
**Transmission**	Vector	Aphids	Aphids	Whitefly, Leafhopper, Tree- hopper, Aphid	Fecal, oral
	Tissue tropism	Phloem	Phloem	Phloem, Mesophyll	Thymocytes, erythroblastoid cells, embryonal tissues
**Infection**	Host Range	Plants families: *Fabaceae, Caricaceae, Solanaceae*	Plants family: *Musaceae Zingiberaceae*	Plants families: *Fabaceae, Caricaceae, Solanaceae, Convolvulaceae*	Animal families: Birds, pigs, freshwater fish Dogs and humans
	Symptoms	Yellows, stunting, mosaic, leaf rolling	Streak mosaic, bushy appearance	Chlorosis, stunting, curling, leaf curling, mottling, leaf distortion	Enlarged lymph nodes, difficulty in breathing, diarrhea, pale skin, jaundice

## Multicellular Way of Life for *Nanoviridae*

In viruses, multipartitism may exert benefits by conferring greater stability due to the genome compartmentalization of smaller-sized segments (Ojosnegros et al., 2011), by increasing the possibility of faster replication of small genomic segments (Nee, 1987), by generating non-mutated offspring (Pressing and Reanney, 1984), or by increasing genome shuffling (Chao, 1988, 1991). In contrast, multipartitism has drawbacks, such as the necessity to either package all the segments together or to ensure the co-entry of an ensemble of virus particles containing at least one copy of each genomic segment, (Chao, 1988; Escriu et al., 2007; Ojosnegros et al., 2011). Moreover, there are serious challenges regarding certain features of these viruses that should be fully elucidated, such as replication, genetic expression, genome encapsidation, method of localization within host cells, transport system (i.e., within-host cell-to-cell or long-distance spread), transmission patterns from one host to another by insect vectors, and evolution of multipartite viruses. Recently efforts were made to understand the intriguing multicellular way of life nanoviruses, but it has been demonstrated thus far only one species of the genus *Nanovirus* i.e., FBNSV, not all species of the family *Nanoviridae*.

## Gene Expression and Viral Infection

Multipartite viruses have a set of 8–10 nucleic acid segments, each encapsidated separately. Each segment has a specific number in the host cell to ensure infection. Some viral genes accumulate at low frequency, whereas others dominate at a high frequency (Sicard et al., 2013). This copy number variation in specific genes within individual cells may considerably affect gene expression in most of the organisms (Stranger et al., 2007; Hastings et al., 2009). Each ssDNA segment accumulates in a reproducible manner with a specific relative copy number in a specific host. These copy numbers, each associated with a specific segment, are defined as the “genome formula” and have proved to be specific to the host plant species. The genome formulae in two different host species i.e., *Vicia faba* and *Medicago truncatula* showed clear variations in the relative frequencies of the eight FBNSV segments calculated in within-host viral populations (Sicard et al., 2013). Although the discovery of the genome formula is remarkable in the biology of multipartite viruses, certain gaps need to be addressed; whether the genome formula is also controlled in the same manner as that in other multipartite viruses, whether it has a role in genetic and phenotypic expression, and whether it is an adaptive and evolvable trait.

The mechanism by which multipartite viruses manage to efficiently infect individual cells with all their segments with whole-genome information is a long-standing mystery. Initially, two possibilities were considered: (i) the particles could penetrate the cells massively with any probability independent of the identity of the contained segment, and (ii) multipartite viruses could somehow sort the particles that enter a cell depending on the encapsidated segment and promote the selective entry of the complete set of the viral genetic information (Sicard et al., 2013, 2016; Dall’ara et al., 2016). This mystery was solved by localizing and quantifying the genome segments of a nanovirus in host plant tissues. It was identified that the segments rarely co-occurred within individual cells; instead, distinct segments accumulated independently in different cells, and that the viral system was functional through complementation across cells (Sicard et al., 2016). These findings deviate from the classical conceptual framework in virology and suggest that various viral particles can localize themselves in separate neighboring cells to produce infection at a multicellular tissue level, thus revealing that the collective presence of all viral genomes in a particular cell is not the basis for infectivity (Sicard et al., 2019). However, these findings are limited to only one nanovirus species i.e., FBNSV. Whether all nanovirus behave like FBNSV is still a question yet to be answered.

## Short and Long Distance Movement

Generally, monopartite viruses transfer their genome information either when moving from cell-to-cell or across long distances to systemically colonize their host. In contrast, multipartite viruses bundle their genetic information in separate virus particles, which must somehow come together to cause infection, as viral trafficking within the host plant is multifarious (Hipper et al., 2013). Three models of movements within the host have been suggested in both monopartite and multipartite viruses. Some plant virus species demonstrate both cell-to-cell movement and movement across long distances as mature virus particles. Some can move from cell-to-cell as nucleoprotein complexes (Lazarowitz and Beachy, 1999) but are not capable of long-distance movement because of their inability to assemble into mature virus particles. Finally, some viral species can spread both by cell-to-cell movement and movement in the plant vasculature as nucleoprotein complexes even without containing the protein coat (Carluccio and Stavolone, 2014). There exists a considerable gap in the literature on the differentiation between multipartite virus movement mechanisms, including those of *Nanoviridae* members and other viruses, however, for multipartite viruses, it is predicted that the multiplicity of cellular infection (MOI) should reach very high values (up to hundreds) for the maintenance of genome integrity (Iranzo and Manrubia, 2012). Owing to the lack of data regarding the movement of multipartite viruses, more investigation is needed with focus on species with multiple segments.

## Vector Transmission

Transmission of viruses, either monopartite or multipartite, mostly requires a particular insect vector (Goodman, 1977; Hogenhout et al., 2008; Hull, 2014; Sicard et al., 2015). In the case of *Nanoviridae* members, aphids transmit nanoviruses and babuviruses (Vetten et al., 2005; Almeida et al., 2009; Sicard et al., 2015), while *Myndus tiffany*, a planthopper, transmits CFDV and is considered a major factor in categorizing CFDV as a separate, unassigned species (Gronenborn et al., 2018). No reports regarding the transmission of *Nanoviridae* members either mechanically or through seeds exist to date because of their restriction to the phloem of infected host plants (Grigoras et al., 2018). Babuviruses are transmitted through finite aphid vectors *Pentalonia nigronervosa* and *Micromyzus kalimpongensis* (Almeida et al., 2009; Bressan and Watanabe, 2011; Ghosh et al., 2015; Halbert and Baker, 2015; Qazi, 2016; Figure 4E). In contrast, nanoviruses can be transmitted by various aphid species, in particular *Aphis craccivora*, *A. gossypii*, *Acyrthosiphon pisum*, *Myzus persicae*, and *Macrosiphum euphorbiae* are the most effective vector species for nanoviruses (Sano et al., 1998; Sicard et al., 2015; Gaafar et al., 2016; Richet et al., 2019; Figure 4F). Among these aphid species, *A. craccivora* is the most abundant and efficient vector, which transmits MDV, SCSV, FBNYV and FBNSV from plant to plant (Franz et al., 1998; Sicard et al., 2015; Gallet et al., 2018; Webster et al., 2018). Some nanoviruses are transmitted by more than one aphid species, but transmission efficiency varies accordingly, e.g., *A. craccivora* transmits SCSV more efficiently than *M. persicae* (Franz et al., 1998; Sicard et al., 2015). In this way, these nanoviruses can have far-reaching effects outside their pivoting areas.

## Translocation of Nanoviruses Within Aphid Vectors

Similar to luteoviruses and geminiviruses, nanoviruses are transmitted in a circular non-propagative manner in their insect vectors (Hogenhout et al., 2008). Virus particles acquired from the infected plant need to cross from the aphids’ gut into the hemolymph; within the hemolymph they are transported to the salivary glands (Blanc et al., 2014), followed by injection into new plants during probing. To ensure successful passage of the integral genome to a new host plant, especially in the case of multipartite viruses such as nanoviruses, it is assumed that at least one functional particle of each type must be transmitted (Iranzo and Manrubia, 2012). Several factors e.g., the accumulation of distinct genome segments at different frequencies (Sicard et al., 2013; Sánchez-Navarro et al., 2013), the stability variations within the host plants (Vaughan et al., 2014), along with the degradation and the relative frequency changes in the segments during the passage within the insect vectors (Sicard et al., 2015) and the impacts of transmission-related bottlenecks (Gallet et al., 2018) may result in the loss of genetic information. It was a highly contentious issue that how the most labile particles can be transmitted as efficiently as the others.

Franz et al. (1999) proposed an aphid helping factor to facilitate virus transport which was confirmed and recognized as NSP (Grigoras et al., 2018). Recently, its potential function investigated as distinct proteins and genome segments of the nanovirus FBNSV were remarkably monitored during transcytosis through the gut and salivary gland cells of its aphid vector *Acyrthosiphon pisum* using a combination of fluorescence *in situ* hybridization and immunofluorescence (Di Mattia et al., 2020). FBNSV follows a route similar to that of the geminiviruses but distinct from that of the luteoviruses, as demonstrated by transportation through cells of the anterior midgut and principal salivary gland. A large number of virus particles enter each susceptible cell to keep distinct genome segments together (Di Mattia et al., 2020; Gaafar and Ziebell, 2020). Previously, similar studies were conducted to track the BBTV (genus *Babuvirus*) within its aphid vector by monitoring the coat protein (Bressan and Watanabe, 2011; Watanabe and Bressan, 2013; Watanabe et al., 2016).

## Role of Nuclear Shuttle Protein in the Transmission

Vector transmission of nanoviruses requires a viral factor or a helper component in addition to the virus particles (Franz et al., 1999). DNA N is the most variable genome component among all components of nanovirus (FBNSV) (Grigoras et al., 2010b). The FBNSV was reconstituted successfully as a fully infectious and sustainably insect-transmissible nanovirus from its multiple cloned DNAs (Grigoras et al., 2009). Recently, the preclusion of aphid transmission was observed when the agroinfectious clones of all segments of FBNSV, except the segment N, were inoculated in a plant though this plant showed similar symptoms as plants that were inoculated with all the eight viral components (Grigoras et al., 2018). The virions that were produced within the plants inoculated with the seven components, excluding DNA-N, abolished the aphid transmission as well when a mutated NSP with a 13-amino acid tag at the carboxyl-terminus was introduced but restored aphid transmission with the introduction of DNA-N of another nanovirus PNYDV (Grigoras et al., 2018), which reinforced the mandatory role of NSP in viral accumulation into the gut cells of the aphid. Co-localization of NSP and coat protein with other viral genome segments, suggest that NSP-virus particle complexes are the viral form that cycles within the aphid body (Di Mattia et al., 2020; Gaafar and Ziebell, 2020). Vector transmission is the major and best-documented mode of transmission of plant viruses, but many gaps i.e., purpose of self interactions between NSPs during translocation, impact of the changes in virus formulas on virus transmission etc., need to be addressed.

## Phylogenetic Analysis of CP and Rep

**FIGURE 5:**
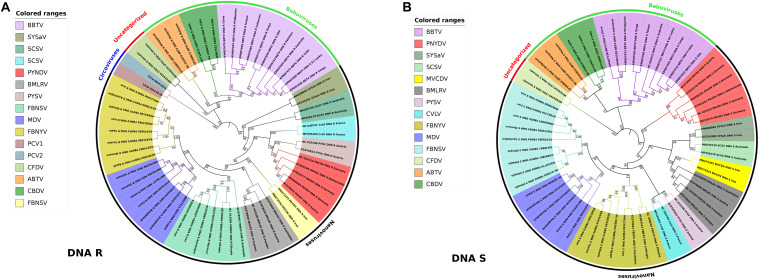
Phylogenetic analysis of DNA-R and DNA-S detected from *Nanoviridae* members. Phylogenetic relationships were generated using the iTOL software. Nevick file for iTOL was generated using MEGA7 program. Virus abbreviations: *Subterranean clover stunt virus* (SCSV), *Black medic leaf roll virus* (BMLRV), *Faba bean necrotic yellows virus* (FBNYV), *Milk Vetch Dwarf Virus* (MDV), *Faba bean necrotic stunt virus* (FBNSY), *Pea yellow stunt virus* (PYSV), *Pea necrotic yellow dwarf virus* (PNYDV), *Cow vetch latent virus* (CvLV), *Sophora yellow stunt-associated virus* (SYSaV), *Milk vetch chlorotic dwarf virus* (MVCDV), *Banana bunchy top virus* (BBTV), *Abaca bunchy top virus* (ABTV), *Cardamom bushy dwarf virus* (CBDV), and *Coconut foliar decay virus* (CFDV). **(A)** Phylogenetic tree of DNA-R generated from around 60 complete genome segments of DNA-R of all members of *Nanoviridae*. Circoviruses: *Porcine circovirus* 1 (PCV1), *Porcine circovirus* 2 (PCV2) were also analyzed due to similarity with Rep protein of nanoviruses. **(B)** Phylogenetic tree of DNA-S generated from around 60 complete genome segments of DNA-S of all members of *Nanoviridae*.

Little has been known about the variability and molecular evolution of nanoviruses. The nucleotide substitution rate of 1.78 × 10^–3^ substitutions per nucleotide per year was observed in FBNSV (Grigoras et al., 2010b) whereas 1.4 × 10^–4^ substitutions per nucleotide per year was determined in local evolution of BBTV in Hawaii (based only on a single base change) (Almeida et al., 2009). Phylogenetic relationships and pairwise sequence identity calculations also depict the linkages and variations among various types of viruses in a better way (Howarth and Vandemark, 1989). In our study, phylogenetic analysis of DNA R (Rep protein) and DNA S (coat protein) was conducted using different nanovirus and babuvirus species. Master Rep is the most similar segment in nanoviruses as well as in babuviruses and is responsible for replication while CP plays a key role in many steps of the infection cycle i.e., translation, targeting of the viral genome to its site of replication, cell-to-cell and/or systemic movement of the virus, symptomatology and virulence of the infection etc., to ensure viral infection (Bol, 2008). Phylogenetic relationships were analyzed using the iTOL (Letunic and Bork, 2019). The Nevick file for iTOL was generated using the MEGA7 program where the multiple sequence alignment tool MUSCLE used to align all sequences. Genome segments of about 60 reported species i.e., both nanoviruses and babuviruses were analyzed. In case of DNA R, circoviruses were taken into consideration as well owing to their similarity with the Rep protein of nanoviruses (Gibbs and Weiller, 1999; Figures 5A,B). Phylogenetic analysis reveals that each species members share same clade in both cases i.e., DNA R and DNA S due to high similarity within the species members than other species members. As, the isolates of each virus were grouped among virus species, and it was confirmed that nanoviruses and babuviruses were also clearly distinguished.

## *Nanoviridae* as an Economic Threat

Agriculture is assumed to be one of the sectors most vulnerable to plant viruses, owing to the potential of viruses to affect plants on a significant scale. Agriculture is considered a fundamental pillar in the world economy and society at large, as it remains a key sector in food supply and is a major source of income. According to the Food and Agriculture Organization of the United Nations, estimated crop losses due to pathogens including plant viruses have been reported to be 20–40% at national and regional levels. Severe damage by plant viruses as well as other pests can significantly decrease the yield of crops as well as their quality, resulting in compromised financial returns due to less produce and lower quality. Plant virus outbreaks, specifically those due to *Geminiviridae* members, have already proven to be a huge setback to the economy in different countries. However, the substantial and devastating consequences of emerging *Nanoviridae* members on crops (Johnstone and Mclean, 1987; Hull, 2014), and the associated economic and social impacts, have largely been underestimated in the agricultural world. In the early 2000s, a sporadic outbreak of FBNYV was reported in Spain (Ortiz et al., 2006). In 2016, a survey of 33 symptomatic faba bean sites in central Germany was conducted toward the end of the growing season to analyze the suspected virus spectrum, and PNYDV was found as the major causal agent in all the sites. A close relationship was observed between PNYDV abundance, symptom-intensity, and a corresponding yield decline in grain weight and crude protein. Combining the relative yield level for each symptom category with its respective appearance, the overall yield gap at the field scale was extrapolated to 4.1 and 9.2% for grain yield and 3.9 and 1.2% for crude protein (Saucke et al., 2019). Furthermore, in the epidemic that occurred in central Germany in 2016, the focal appearance with a blackish core can be regarded as a PNYDV-specific feature for temperate faba beans (Ziebell, 2017; Saucke et al., 2019).

Considering the importance of host species infected by babuviruses and nanoviruses categorically may help in the development of broad-scale adversary agents. Bananas are among the top 10 food plants, especially valued in the tropics and are the source of staple food, nutrition and income for millions of banana farmers worldwide. These factors have led to its high demand and the ultimate increase in production over the last decade. BBTV outbreaks alone across 2007–2010 in different states of India caused production losses of at least US$50 million (Balasubramanian and Selvarajan, 2014). *Vicia faba*, a bean family member found in the Middle East, the Mediterranean region, China, and Ethiopia, is a multipurpose crop used for both food and fodder (hay, silage, and straw). More than 50 species of the bean family are used in human food production as seeds, as an important livestock feed, and for economic benefit owing to their value and consideration as a cash crop in Egypt and Sudan. Along with bean family members, recent developments clearly outline the expansion in the host range of nanoviruses (as mentioned above). Among these new hosts, papaya, tomato and pepper are the most important ones (though parsley is a valuable member as well). Papaya (*C. papaya* L.) is the third most cultivated fruit in the world and found in both tropical and subtropical zones. It is produced in about 60 countries, mainly in developing countries, with an estimated production of 11.22 Mt (annual growth rate 4.35 percent between 2002 and 2010). The high nutritional and medicinal content of papaya make it an attractive crop for farmers to grow. Tomato (*S. lycopersicum*) is an extremely important Solanaceae member along with pepper (*C. annum*); both are produced and consumed by people across the world and are used in many cuisines worldwide. In 2017, the worldwide production of tomatoes totaled 170.8 million tons, while pepper production was 576,949 tons in 2018. These high numbers of production are directly and indirectly connected with food security and economic growth in the countries that produce them. Thus, one can speculates that nanoviruses might have the ability for significant impact on these newly reported food crops by affecting their yield (Grigoras et al., 2008; Rosario et al., 2012; Lal et al., 2018, 2020). So, expanding host range threatens to develop into unexpected and serious epidemics but this prediction is still somewhat obscure.

## Future Challenges Regarding *Nanoviridae*

Recent developments highlight the diversity in host ranges of the *Nanoviridae* members, with strong evidence indicating that an increasing number of host species will be reported with time. Along with *Geminiviridae*, *Nanoviridae* members also play a notable role in the plant world, which necessitates equal attention to geminiviruses in understanding their complexities. Particularly, their mode of infection, method of localization, evolutionary history, host ranges, multicellular way of life, preferred hosts and environment, and transmission pattern are all features which remain to be investigated in detail. The agriculture sector has a new emerging threat of *Nanoviridae* infection. It is the collective responsibility of the scientific community to develop a thorough plan and policy to counteract this before a devastating effect on food security and the global economy is realized.

## Author Contributions

AL, E-JK, and SL outlined and conceptualized the review theme. AL wrote the first draft of the manuscript. J-KK, TV, IS, PH, E-JK, and SL contributed to manuscript preparation and revision, and also read and approved the submitted version. All authors contributed to the article and approved the submitted version.

## Conflict of Interest

The authors declare that the research was conducted in the absence of any commercial or financial relationships that could be construed as a potential conflict of interest.
